# Dynamics of hemoglobins during nodule development, nitrate response, and dark stress in *Lotus japonicus*

**DOI:** 10.1093/jxb/erad455

**Published:** 2023-11-17

**Authors:** Samuel Minguillón, Ángela Román, Carmen Pérez-Rontomé, Longlong Wang, Ping Xu, Jeremy D Murray, Deqiang Duanmu, Maria C Rubio, Manuel Becana

**Affiliations:** Departamento de Biología Vegetal, Estación Experimental de Aula Dei, Consejo Superior de Investigaciones Científicas (CSIC), Avenida Montañana 1005, Zaragoza, and Unidad Asociada GBsC (BIFI-Unizar) al CSIC, Zaragoza, Spain; Departamento de Biología Vegetal, Estación Experimental de Aula Dei, Consejo Superior de Investigaciones Científicas (CSIC), Avenida Montañana 1005, Zaragoza, and Unidad Asociada GBsC (BIFI-Unizar) al CSIC, Zaragoza, Spain; Departamento de Biología Vegetal, Estación Experimental de Aula Dei, Consejo Superior de Investigaciones Científicas (CSIC), Avenida Montañana 1005, Zaragoza, and Unidad Asociada GBsC (BIFI-Unizar) al CSIC, Zaragoza, Spain; National Key Laboratory of Agricultural Microbiology, College of Life Science and Technology, Huazhong Agricultural University, Wuhan 430070, China; CAS-JIC Centre of Excellence for Plant and Microbial Science, Centre for Excellence in Molecular Plant Sciences, Shanghai Institute of Plant Physiology and Ecology, Chinese Academy of Sciences, Shanghai, China; CAS-JIC Centre of Excellence for Plant and Microbial Science, Centre for Excellence in Molecular Plant Sciences, Shanghai Institute of Plant Physiology and Ecology, Chinese Academy of Sciences, Shanghai, China; National Key Laboratory of Agricultural Microbiology, College of Life Science and Technology, Huazhong Agricultural University, Wuhan 430070, China; Departamento de Biología Vegetal, Estación Experimental de Aula Dei, Consejo Superior de Investigaciones Científicas (CSIC), Avenida Montañana 1005, Zaragoza, and Unidad Asociada GBsC (BIFI-Unizar) al CSIC, Zaragoza, Spain; Departamento de Biología Vegetal, Estación Experimental de Aula Dei, Consejo Superior de Investigaciones Científicas (CSIC), Avenida Montañana 1005, Zaragoza, and Unidad Asociada GBsC (BIFI-Unizar) al CSIC, Zaragoza, Spain; University of Warwick, UK

**Keywords:** Gene regulation, knockout mutants, legume nodule senescence, *Lotus japonicus*, plant hemoglobins, symbiosis

## Abstract

Legume nodules express multiple leghemoglobins (Lbs) and non-symbiotic hemoglobins (Glbs), but how they are regulated is unclear. Here, we study the regulation of all Lbs and Glbs of *Lotus japonicus* in different physiologically relevant conditions and mutant backgrounds. We quantified hemoglobin expression, localized reactive oxygen species (ROS) and nitric oxide (NO) in nodules, and deployed mutants deficient in Lbs and in the transcription factors NLP4 (associated with nitrate sensitivity) and NAC094 (associated with senescence). Expression of Lbs and class 2 Glbs was suppressed by nitrate, whereas expression of class 1 and 3 Glbs was positively correlated with external nitrate concentrations. Nitrate-responsive elements were found in the promoters of several hemoglobin genes. Mutant nodules without Lbs showed accumulation of ROS and NO and alterations of antioxidants and senescence markers. NO accumulation occurred by a nitrate-independent pathway, probably due to the virtual disappearance of Glb1-1 and the deficiency of Lbs. We conclude that hemoglobins are regulated in a gene-specific manner during nodule development and in response to nitrate and dark stress. Mutant analyses reveal that nodules lacking Lbs experience nitro-oxidative stress and that there is compensation of expression between Lb1 and Lb2. They also show modulation of hemoglobin expression by NLP4 and NAC094.

## Teaser

Legume hemoglobins are regulated in a gene-specific manner during nodule development and in response to nitrate and dark stress; nodules lacking leghemoglobins experience nitro-oxidative stress. Hemoglobin expression is modulated by NLP4 and NAC094.

## Introduction

Hemoglobins are widespread in archaea, bacteria, and eukaryotes, showing an outstanding diversity of structures and functions (see reviews by Vinogradov *et al.*, 2005; Hoy and Hargrove, 2008; Becana *et al.*, 2020). Symbiotic hemoglobins seem to be restricted to the nodules of legumes and actinorhizal plants. Their main function is to transport O_2_ in the cytosol of infected cells to the N_2_-fixing bacteria at a steady low concentration compatible with nitrogenase activity and bacterial respiration (Appleby, 1984; Ott *et al.*, 2005; Wang *et al.*, 2019). The symbiotic hemoglobins of legumes (leghemoglobins; Lbs) are present in nodules at millimolar concentrations as a mixture of multiple isoproteins or components encoded by different genes (Larrainzar *et al.*, 2020). Plants also express non-symbiotic hemoglobins (phytoglobins; Glbs) at (sub)micromolar concentrations in virtually all organs, from roots and leaves to flowers and fruits (Hill, 2012). Glbs are categorized into three classes according to phylogeny and biochemical properties. Class 1 Glbs have an extremely high O_2_ affinity and are therefore unlikely to transport and deliver O_2_. They contribute to the survival of plants under hypoxic conditions and to the homeostasis of nitric oxide (NO), a key signaling gas molecule with a plethora of functions (Domingos *et al.*, 2015). Class 1 Glbs are also critical for the onset and functioning of the legume–rhizobia symbiosis by modulating NO levels (Shimoda *et al.*, 2005; Fukudome *et al.*, 2016; Berger *et al.*, 2020). Class 2 Glbs show homology with Lbs, moderate O_2_ affinity, and NO-scavenging activity (Hebelstrup and Jensen, 2008; Smagghe *et al.*, 2009). Class 3 Glbs share homology with the ‘truncated’ hemoglobins of prokaryotes, exhibit low O_2_ affinity, and have unknown functions (Vieweg *et al.*, 2005).

The major aim of this work was to investigate the regulation of the whole set of hemoglobins in a leguminous plant during nodule development and in response to nitrate and dark stress conditions. For this purpose, it was essential to individualize all Lb and Glb mRNAs and proteins. We chose the model legume *Lotus japonicus* because it is more amenable for this study than *Medicago truncatula*, which contains 12 Lbs and five Glbs (Berger *et al.*, 2020; Larrainzar *et al.*, 2020), and also because we wanted to investigate two unusual Glbs, Glb2-1 and Glb2-2, tentatively assigned to class 2 Glbs. This assignment is based on a number of differences of those Glbs with respect to genuine Lbs, albeit they do not seem to be either typical class 2 Glbs like those of Arabidopsis, tomato, and sugar beet (Becana *et al.*, 2020). The genome of *L. japonicus* contains nine hemoglobin genes that encode three Lbs, two class 1 Glbs, two class 2 Glbs, and two class 3 or ‘truncated’ Glbs. The first part of our study was focused on the regulation of hemoglobins by deploying mutants that differ in Lb composition (Wang *et al.*, 2019). The second part of the study examined the expression of all the hemoglobins in nodules induced to senesce by treating plants with nitrate or exposing them to dark stress. For this we used wild-type (WT) and mutant plants defective in the transcription factor NODULE INCEPTION-LIKE PROTEIN 4 (NLP4), previously known as NITRATE UNRESPONSIVE SYMBIOSIS 1 (NRSYM1), or in the transcription factor NAC094. These transcription factors were selected because NLP4 is related to the nitrate response of the symbiosis (Nishida *et al.*, 2018) and NAC094 is associated with nodule senescence (Wang *et al.*, 2023). Here, we show that hemoglobins of *L. japonicus* are distinctly regulated during development as well as in the nitrate and dark stress responses of nodules. Furthermore, our results with loss-of-function mutants demonstrate that Lb-deficient nodules experience nitro-oxidative stress and provide insights into how the expression of the various Lb and Glb isoproteins is modulated by NLP4 and NAC094.

## Materials and methods

### Plant material and growth conditions

Single (*lb3*), double (*lb13* and *lb23*), and triple (*lb123*) *lb* mutants of *Lotus japonicus* ecotype MG-20 were generated using CRISPR/Cas9 [clustered regularly interspaced palindromic repeats (CRISPR)/CRISPR-associated protein 9] genome editing (Wang *et al.*, 2019). Mutant plants of ecotype MG-20 deficient in NLP4 or NAC094 were isolated by chemical mutagenesis (Nishida *et al.*, 2018) or by CRISPR/Cas9 (Wang *et al.*, 2023), respectively. Seeds were germinated and seedlings were grown in plates following standard protocols (Villar *et al.*, 2021) in a cabinet with 23 °C/21 °C (day/night), 120–140 μmol photons m^−2^ s^−1^, and a 16 h photoperiod. Seedlings were then transferred to vermiculite-containing pots, nodulated with *Mesorhizobium loti* strain MAFF303099 (1.5 × 10^8^ cells per seedling), and grown in cabinets, being watered twice a week with N-free B&D nutrient solution (Broughton and Dilworth, 1971). For nitrate treatment, plants at 4 weeks post-inoculation (wpi) were supplied for 2 d with B&D solution containing 0 (control), 0.5, 5, or 10 mM KNO_3_ as indicated in each experiment. For dark stress, nodulated plants were exposed to continuous darkness, for the number of days indicated in each experiment, in growth cabinets under otherwise identical conditions. Nodules were harvested in liquid nitrogen and stored at –80 °C until use.

### Gene expression

Total RNA was extracted from nodules using the RNAqueous Total RNA isolation kit and was treated with DNase I (Thermo Fisher Scientific). cDNA was synthesized with MMLV reverse transcriptase (Promega). Quantitative reverse transcription–PCR (qRT–PCR) analyses were performed using a QuantStudio3 Real-Time PCR System (Applied Biosystems) as described (Rubio *et al.*, 2019). Gene identifiers, primer sequences, and amplification efficiencies are provided in Supplementary Table S1, and gene exon–intron composition and primer positions are given in Supplementary Fig. S1. The amplification efficiency for each set of primers was calculated by analysis of standard curves obtained from serial template dilutions (Pfaffl, 2001). Normalized relative quantities (NRQ) were calculated using the geometric means of two reference genes (Rubio *et al.*, 2019), either *LjUbiquitin* and *LjATP synthase* or *LjUbiquitin* and *LjeIF4A*. The selection of the reference gene pairs was made based on their stability in each experiment.

### Immunoblots

Immunoblots were carried out on 15% native-PAGE or 15% SDS–PAGE gels following standard protocols. The primary antibodies were generated against common bean Lb*a* and *L. japonicus* Glb1-2, Glb2-1, and Glb3-2, and were affinity purified (Sainz *et al.*, 2013, 2015). Ferritin antibody was generated against Arabidopsis ferritin 1 and was kindly provided by Brigitte Touraine (University of Montpellier, France). The secondary antibody was goat anti-rabbit IgG conjugated with horseradish peroxidase. Further details are indicated in the figure legends. Immunoreactive proteins were detected by chemiluminescence using the SuperSignal West Pico kit (Pierce) and images were acquired using a ChemiDoc MP Imaging System (Bio-Rad).

### Detection of reactive molecules

ROS and NO were localized in nodule sections (~85 µm) by cytochemical staining. Superoxide radical production was detected using nitroblue tetrazolium (NBT). Nodule sections were immersed in staining buffer (50 mM potassium phosphate, pH 7.8, 0.25 mM NBT) for 1 h at room temperature in the dark. For H_2_O_2_ detection, nodule sections were stained with 3,3'-diaminobenzidine (DAB) in buffer (10 mM Tris–HCl, pH 7.4, 1 mg ml^–1^ DAB) for 5 min in the dark at room temperature. For NO detection, nodule sections were incubated with 10 µM 4-amino-5-methylamino-2',7'-diﬂuoroﬂuorescein diacetate in buffer (10 mM Tris–HCl, pH 7.4, 10 mM KCl) for 30 min at room temperature in the dark. To test inhibitors and/or scavengers, nodule sections were pre-incubated for 1 h (ROS) or 30 min (NO) in the dark, then subjected to the same staining protocol as described above but keeping the inhibitors during the incubation period. Specifically, we examined the effects of diphenyleneiodonium (100 µM DPI) on NBT staining; KI (10 mM) on DAB staining; and 2-(4-carboxyphenyl)-4,4,5,5-tetramethylimidazoline-1-oxyl-3-oxide (1 mM cPTIO) on NO staining. DPI is an inhibitor of NADPH oxidase, KI is a H_2_O_2_ scavenger; and cPTIO is an NO scavenger (Dunand *et al.*, 2007; Sandalio *et al.*, 2008). Images of ROS were acquired using a DM750 (Leica) light microscope and images of NO with an LSM880 (Zeiss) confocal microscope with excitation at 488 nm and detection at 498–549 nm.

### Determination of antioxidant enzyme activities

Enzymes were extracted from nodules in a medium consisting of 50 mM potassium phosphate buffer (pH 7.8), 0.1 mM EDTA, 1% (w/v) soluble polyvinylpyrrolidone, and 0.1% (v/v) Triton X-100. Nodule extracts were centrifuged at 13 000 *g* for 10 min at 4 °C, and superoxide dismutase (SOD), catalase, and ascorbate peroxidase (APX) activities were determined in the supernatants using standard protocols. Total SOD activity was measured following the inhibition of the reduction of ferricytochrome *c* by superoxide radicals generated by a xanthine–xanthine oxidase system (Rubio *et al.*, 2001). The SOD isoforms were resolved on native-PAGE gels stained with NBT and were identified by in-gel differential inhibition with 3 mM KCN (inhibitor of CuZnSOD) and 5 mM H_2_O_2_ (inhibitor of CuZnSOD and FeSOD). Further identification of SOD isoforms was based on previous studies (Rubio *et al.*, 2007). The activity of each isoform was assessed by calculating the relative proportions by gel densitometry and referring those values to the total SOD activity of extracts. Catalase activity was assayed by following the decomposition of H_2_O_2_ at 240 nm (Aebi, 1984), and APX activity by following ascorbate oxidation at 290 nm (Asada, 1984). All enzyme activities were measured at 25 °C within a linear range.

### Search for nitrate-responsive elements and double nitrate-responsive elements in hemoglobin promoters

The promoters of *L. japonicus* hemoglobin genes were searched using MEME (https://meme-suite.org/meme/). To sensitively detect nitrate-responsive elements (NREs) in the target promoters, they were analyzed along with nitrite reductase (*NiR*) genes of 38 different plant species (–500 bp to ATG) that were previously shown to contain canonical NREs. The sequence list can be found in Bailey *et al.* (2006) and Jiang *et al.* (2021). To detect double nitrate-responsive elements (dNREs) in the *L. japonicus* hemoglobin promoters, they were analyzed along with 12 *Lb* genes of *M. truncatula* that were previously shown to harbor single copies of these elements. The sequence list can be found in Jiang *et al.* (2021). For searching, the minimum and maximum sites per motif used were set at 21–60 bp and 24–50 bp for the dNRE and NRE analyses, respectively. The detected motifs were confirmed as positive matches through TOMTOM (MEME suite) and by comparison with published sequences (Jiang *et al.*, 2021).

## Results

### Gene profiling reveals distinct regulation of each Lb and Glb during the development and nitrate response of nodules

**Fig. 1.**
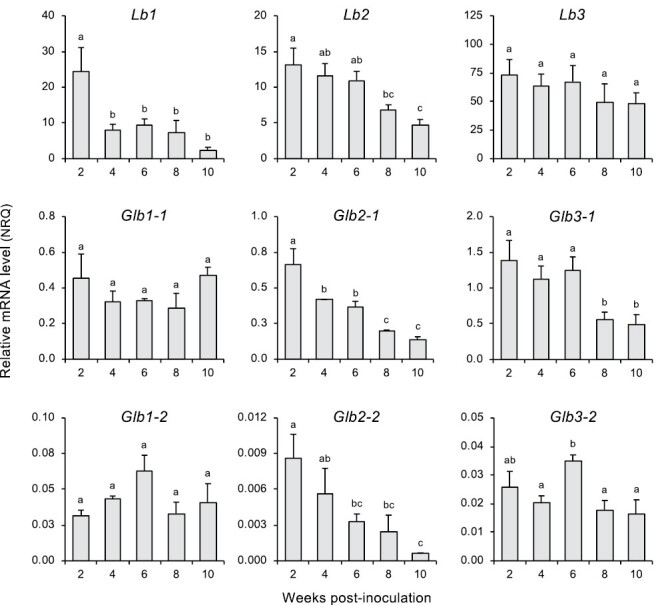
Expression of hemoglobin genes during development and senescence of *L. japonicus* nodules. Transcript levels were normalized using *LjUbiquitin* and *LjeIF4A* as reference genes. Data are given in normalized relative quantities (NRQ) and are means ±SE of 3–5 biological replicates. Different letters indicate significant differences among means according to Duncan’s multiple range test (*P*<0.05).

We first quantified the expression levels of all *L. japonicus* hemoglobin genes during nodule development. Nodules were harvested at 2 wpi (young), 4–6 wpi (mature), and 8–10 wpi (senescent). The data of this and other experiments involving qRT–PCR were calculated as NRQ. To this end, it was critical to design gene-specific primers because the three *Lb* genes share extremely high nucleotide sequence identity in the coding regions: 99% between *Lb1* and *Lb2*, 87% between *Lb1* and *Lb3*, and 87% between *Lb2* and *Lb3.* This problem was circumvented by designing one of the primer pairs in the 5'-untranslated region (UTR) of *Lb2* and in the 3'-UTR of *Lb1* (Supplementary Fig. S1). The hemoglobin genes display distinct expression levels and patterns (Fig. 1). In young nodules, there was a difference of ~8000-fold in the mRNA levels of hemoglobin genes between the most (*Lb3*) and least (*Glb2-2*) expressed. Based on the order of magnitude of expression (NRQ for young nodules in parentheses), we can distinguish four groups of hemoglobins: *Lbs* (13–73); *Glb1-1*, *Glb2-1*, and *Glb3-1* (0.5–1.4); *Glb1-2* and *Glb3-2* (0.03); and *Glb2-2* (0.009). The same groups were observed, albeit with different ranges, when considering the NRQ in senescing nodules. These observations are in line with the high Lb concentrations usually observed in legume nodules (Berger *et al.*, 2020; Larrainzar *et al.*, 2020), as well as with the higher expression of *Glb1-1* and *Glb3-1* relative to their *Glb1-2* and *Glb3-2* counterparts in nodules of *L. japonicus* grown in hydroponics (Bustos-Sanmamed *et al.*, 2011).

**Fig. 2.**
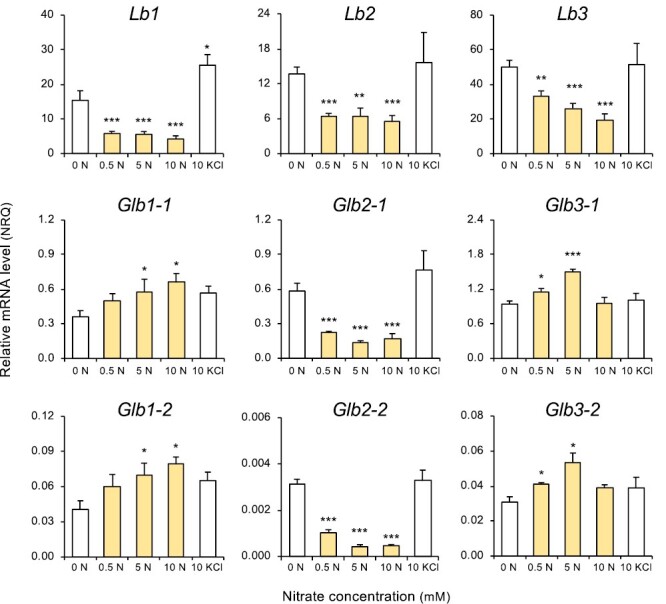
Expression of hemoglobin genes in response to nitrate in *L. japonicus* nodules. Nodulated plants were grown without nitrate until 4 wpi and then were treated with 0, 0.5, 5, or 10 mM KNO_3_ for 2 d. Control plants supplied with 10 mM KCl for 2 d in place of nitrate were also included. Transcript levels were normalized using *LjUbiquitin* and *LjeIF4A* as reference genes. Data are given in normalized relative quantities (NRQ) and are means ±SE of 3–7 biological replicates. Asterisks indicate significant differences compared with the control (0 N) based on Student’s *t*-test (*P<0.05; **P<0.01; ***P<0.001).

The *Lb3* gene was by far the most highly expressed among all hemoglobin genes and did not vary during nodule development (Fig. 1). In contrast, *Lb1* expression was drastically reduced from young to mature nodules, remaining thereafter at a low level, whereas *Lb2* expression decreased gradually with advancing age. There were only slight or no changes in expression of the genes encoding class 1 and 3 Glbs, except *Glb3-1*, which was reduced ~3-fold in senescent nodules. Moreover, *Glb2-1* and *Glb2-2* behaved like *Lb2*, with a progressive reduction in expression from young to senescent nodules (Fig. 1). We also investigated the response of hemoglobin gene expression in nodules of plants that had been growing without nitrate until 4 wpi and then supplied with low or high nitrate for 2 d (Fig. 2). Nitrate concentrations were selected for comparison because 0.5 mM nitrate is non-inhibitory for nodulation, whereas 5 mM and 10 mM nitrate induce nodule senescence (Nishida *et al.*, 2018; Wang *et al.*, 2023). Nitrate decreased expression of Lbs and class 2 Glbs; however, it increased expression of class 1 Glbs gradually from 0.5 mM to 10 mM and of class 3 Glbs at 0.5 mM and 5 mM, returning to the control value at 10 mM. Interestingly, a decline in *Lb* and *Glb2* mRNAs was already apparent at 0.5 mM nitrate relative to nodules without nitrate. Based on the transcriptional response to this low concentration, the most nitrate-sensitive *Lb* gene was found to be *Lb1*, followed by *Lb2* and *Lb3*, with respective decreases of 62, 53, and 34% in mRNA levels. No major differences in the nitrate response were observed between the two members within each class of hemoglobin genes (Fig. 2). The differential expression of hemoglobin genes in response to nitrate delineates a clear-cut separation of Lbs from class 1 and 3 Glbs. However, as occurred with nodule development (Fig. 1), this was not the case for class 2 Glbs, which behave like Lbs (Fig. 2).

### Lb levels are precisely regulated through transcriptional feedback

**Fig. 3.**
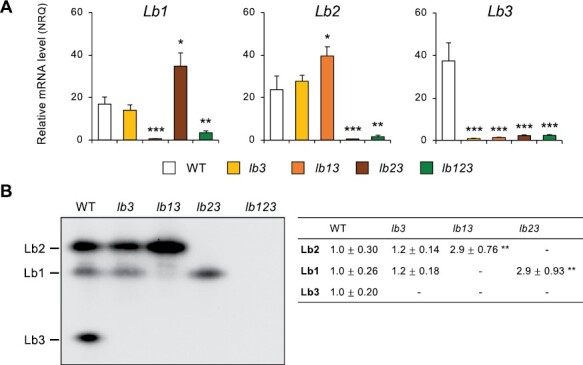
Expression of the three leghemoglobins (Lbs) in nodules of Lb mutants (*lb*) of *L. japonicus*. Nodulated plants were grown without nitrate until 4 wpi. The knockout *lb* mutants were generated by CRISPR/Cas9 and are deficient in one (*lb3*), two (*lb13* and *lb23*), or all three (*lb123*) Lbs. (A) Transcript levels were normalized using *LjUbiquitin* and *LjATP synthase* as reference genes and are given in normalized relative quantities (NRQ). Data are means ±SE of 3–5 biological replicates. Asterisks indicate significant differences with respect to the WT plants based on Student’s *t*-test (*P<0.05; **P<0.01; ***P<0.001). (B) Immunoblots of native-PAGE gels were used to resolve the three Lbs. Gels were loaded with 25 μg of protein per lane and challenged with an affinity-purified polyclonal antibody raised against common bean Lb*a*. The primary and secondary antibodies were used at dilutions of 1:1000 and 1:80 000, respectively. Each immunoblot is representative of gels loaded with proteins from three different biological replicates per genotype. The right side of (B) shows the densitometric determination (ImageJ) of Lb amounts on gels, which were normalized with respect to those of the WT. Data are means ±SE of three biological replicates, and asterisks denote significant differences based on Student’s *t*-test (**P<0.01). The hyphens indicate that the protein is not detectable.

To gain further insight into regulation of hemoglobin genes, we characterized mutants deficient in one, two, or all three Lbs (Fig. 3). For this study, it was essential to quantify each *Lb* mRNA by qRT–PCR and each Lb protein on immunoblots of native gels. This allowed us to verify the primer specificities and the knockout mutations, as well as to uncover a compensatory phenomenon by which the double mutants overexpress their single functional Lb. Thus, we observed an increase of Lb1 expression (mRNA and protein) in the *lb23* mutant and of Lb2 expression (mRNA and protein) in the *lb13* mutant relative to the WT nodules (Fig. 3). We then phenotyped the nodulated mutants at 4 wpi by measuring growth parameters (Supplementary Fig. S2). At this stage, all plants exhibited an intense green color of leaves, with the exception of *lb123*, which appeared slightly less green, suggesting the start of nitrogen deficiency. The single and double mutants showed decreases in the growth of the shoot (length and weight) and leaves (number and weight) and, in the case of *lb13*, also of the root (length and weight) (Supplementary Fig. S2). The triple mutant showed decreases in all of these parameters except root length. Interestingly, *lb3*, *lb13*, and *lb123* had lower nodule weights per plant than the WT, but *lb123* produced more nodules, probably in an attempt to overcome the lower nitrogen fixation rate observed in this mutant (Wang *et al.*, 2019).

**Fig. 4.**
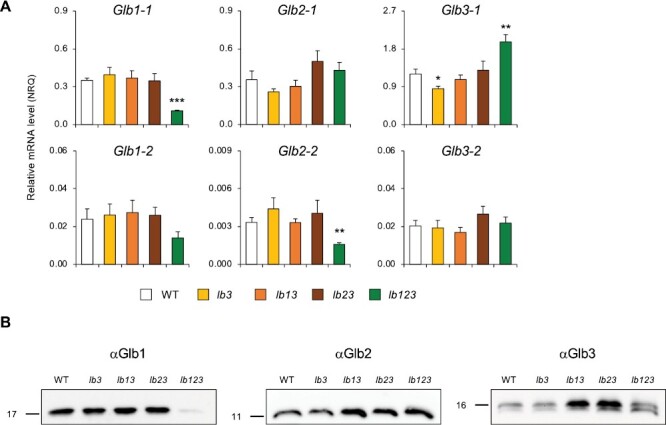
Expression of six phytoglobins (Glbs) in nodules of leghemoglobin mutants (*lb*) of *L. japonicus*. Nodulated plants were grown without nitrate until 4 wpi. The knockout *lb* mutants are indicated in the legend of Fig. 3. (A) Transcript levels are given in normalized relative quantities (NRQ) using *LjUbiquitin* and *LjATP synthase* as reference genes. Data are means ±SE of 3–5 biological replicates. Asterisks indicate significant differences with respect to the WT plants based on Student’s *t*-test (*P<0.05; **P<0.01; ***P<0.001). (B) Immunoblots of SDS–PAGE gels loaded with 10, 20, or 30 μg of protein per lane for Glb1, Glb2, and Glb3, respectively. Proteins were detected with affinity-purified polyclonal antibodies raised against *L. japonicus* Glb1-2 (αGlb1), Glb2-1 (αGlb2), and Glb3-2 (αGlb3). The primary and secondary antibodies were used at dilutions of 1:1000 and 1:80 000, respectively. The immunoblots are representative of gels loaded with proteins from three different biological replicates per genotype. The molecular mass (kDa) is indicated on the left.

The next step was to determine the expression profiles of Glbs (mRNAs and proteins) in nodules (Fig. 4A, B). No major changes were found except for the *lb123* mutant, which showed a lower expression of Glb1-1 (mRNA and protein) and Glb2-2 (mRNA) and a higher expression of Glb3-1 (mRNA and protein) relative to the WT (Fig. 4A, B). The decrease in *Glb2-2* mRNA could not be tested at the protein level because *Glb2-2* is expressed at levels ~100-fold lower than *Glb2-1* (Figs 1, 2, 4). Unexpectedly, we observed in nodule extracts two close immunoreactive bands with the Glb3-2 antibody (Fig. 4B). This antibody recognizes Glb3-1 and Glb3-2 as they share 83% amino acid identity. However, the two protein bands are still present in *glb3-2* knockout mutants but almost disappear in *glb3-1* knockout mutants (Supplementary Fig. S3), indicating that both bands correspond to Glb3-1. These might have originated by alternative splicing or post-translational modification. The enhanced content of both proteins matched the *Glb3-1* mRNA profile in the *lb123* mutant but not in the *lb13* and *lb23* mutants, which showed enhanced protein content with no change in the transcript level (Fig. 4A, B).

### Lb-deficient mutants show alterations in the production of reactive molecules and antioxidant defenses leading to nitro-oxidative stress

**Fig. 5.**
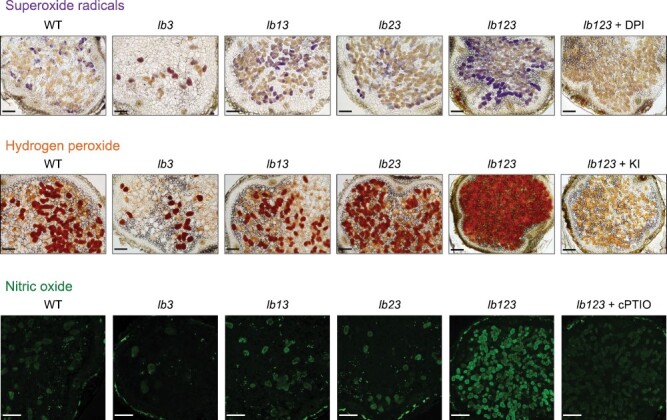
Localization of ROS and NO in nodules of leghemoglobin mutants (*lb*) of *L. japonicus*. Nodulated plants were grown without nitrate until 4 wpi. The production of superoxide radicals was localized using nitroblue tetrazolium staining, which is reduced by superoxide to insoluble blue formazan. A control with 100 µM diphenyleneiodonium (DPI) was included to test the involvement of NADPH oxidases in superoxide generation. The production of H_2_O_2_ was detected by the oxidation of 3,3'-diaminobenzidine to a brown precipitate by endogenous peroxidases. A control with 10 mM potassium iodide (KI), which oxidizes H_2_O_2_, was included to ascertain genuine H_2_O_2_ production. The production of NO was localized using 4-amino-5-methylamino-2',7'-diﬂuoroﬂuorescein diacetate. This cell membrane-permeable probe is hydrolyzed by intracellular esterases and reacts with N_2_O_3_ derived from NO in the presence of O_2_ to yield a highly fluorescent triazole (green). A control with 1 mM 2-(4-carboxyphenyl)-4,4,5,-tetramethylimidazoline-1-oxyl-3-oxide (cPTIO), an NO scavenger, was included to ascertain genuine production of NO. For all three panels, note the higher staining intensity in the nodule sections of the *lb123* mutant and, notably, a lower intensity in those of the *lb3* mutant. For each ROS and NO, three or four nodules per genotype with two sections per nodule were examined. The whole experiment was repeated three times for ROS and twice for NO. Scale bars, 150 µm.

**Fig. 6.**
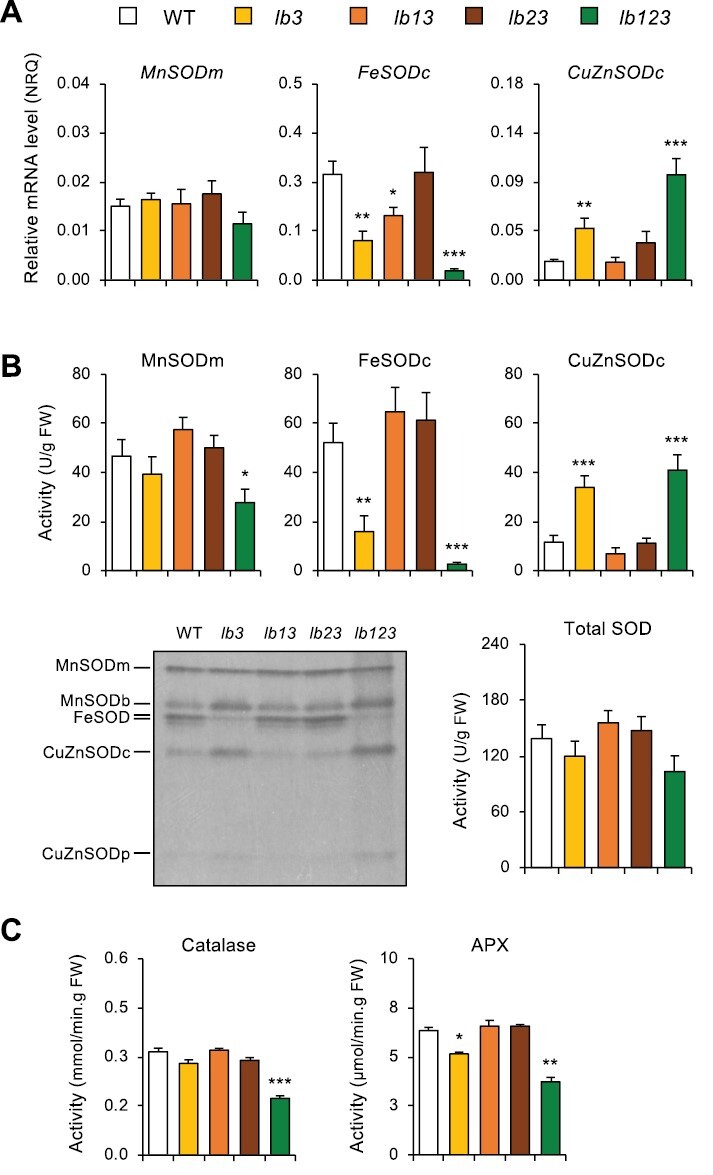
Expression of several major antioxidant enzymes in nodules of leghemoglobin mutants (*lb*) of *L. japonicus*. Nodulated plants were grown without nitrate until 4 wpi. (A) Transcript levels of superoxide dismutase (*SOD*) genes are expressed in normalized relative quantities (NRQ) using *LjUbiquitin* and *LjATP synthase* as reference genes. (B) Total SOD and SOD isoform activities are expressed in units per gram of nodule fresh weight. One SOD unit is defined as the amount of enzyme that inhibits the reduction of ferricytochrome *c* by 50%. (B) also shows an in-gel SOD activity assay (negative image of the gel) revealing the various isoforms: mitochondrial MnSOD (MnSODm), bacteroid MnSOD (MnSODb); FeSOD (two bands: the upper most intense band is the cytosolic isoform; the lower faint band is the plastid isoform); CuZnSODc, cytosolic CuZnSOD; and CuZnSODp, plastid CuZnSOD. (C) Catalase and ascorbate peroxidase (APX) activities are given, respectively, in mmol of H_2_O_2_ decomposed and µmol of ascorbate oxidized per min and gram of nodule fresh weight. For all panels, data are means ±SE of 3–7 biological replicates, and asterisks indicate significant differences relative to the WT based on Student’s *t*-test (*P<0.05; **P<0.01; ***P<0.001).

We next investigated whether Lb deficiencies cause nitro-oxidative stress in nodules by localizing the production of ROS and NO by using cytochemical staining. The production of superoxide radicals, H_2_O_2_, and NO increased consistently in the nodules of *lb123* but not in the nodules of the single and double mutants, indicating that the presence of a single Lb is sufficient to avoid nitro-oxidative stress (Fig. 5). To confirm this, we measured some key antioxidant enzyme transcripts and activities in all the mutant nodules (Fig. 6A, B). The transcript level of each *SOD* gene was quantified by qRT–PCR (Fig. 6A) and the SOD isoforms were in-gel assayed for activity with NBT (Fig. 6B). To calculate the activity of each isoform, densitometric values of abundance were referred to the total SOD activity of nodule extracts. Interestingly, we found major changes in the SOD isoform composition of *lb3* and *lb123* nodules, with an apparent compensation in expression (mRNAs and enzyme activities) of the two cytosolic isoforms. Thus, in the two mutants, there was a decrease in FeSODc and a concomitant increase in CuZnSODc, while the total SOD activity remained constant. Significant changes were also detected in the FeSODc mRNA level of *lb13* and in the mitochondrial MnSOD activity of *lb123* (Fig. 6A, B). We next measured the activities of the two major H_2_O_2_-scavenging enzymes of nodules. Catalase is a peroxisomal enzyme and APX occurs as several isoforms; however, the APX activity assayed in our study corresponds to the cytosolic isoform because it is by far the most abundant in nodules and, unlike the other isoforms, is stable when ascorbate is omitted from the extraction medium (Dalton *et al.*, 1987). We found significant decreases of catalase activity in the *lb123* mutant and of APX activity in the *lb3* and *lb123* mutants (Fig. 6C).

**Fig. 7.**
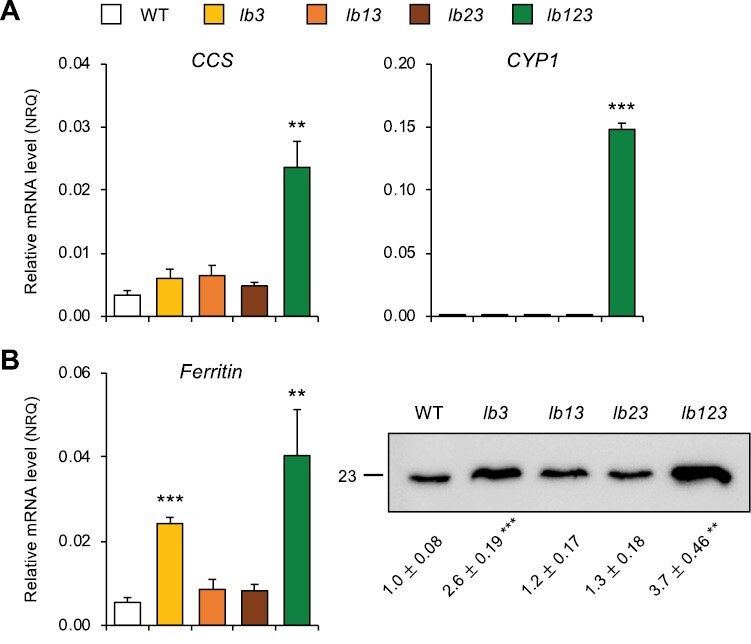
Expression profiles of genes associated with senescence and metal homeostasis in nodules of leghemoglobin mutants (*lb*) of *L. japonicus.* (A) Transcript levels of copper chaperone for CuZnSOD (*CCS*) and cysteine protease (*CYP1*). (B) Transcript and protein levels of ferritin. For both panels, transcript levels are expressed in normalized relative quantities (NRQ) using *LjUbiquitin* and *LjATP synthase* as the reference genes. Data are means ±SE of 3–4 biological replicates. For the immunoblot, proteins (10 μg per lane) were resolved on SDS–PAGE gels and ferritin was detected with an antibody raised against Arabidopsis ferritin 1. The primary and secondary antibodies were used at dilutions of 1:5000 and 1:40 000, respectively. The immunoblot shows the molecular mass (kDa) on the left and the values of the densitometric scan (ImageJ) below each lane. Data are means ±SE of three biological replicates, normalized to the values of the WT nodules. For both panels, asterisks indicate significant differences relative to the WT according to Student’s *t*-test (**P<0.01; ***P<0.001).

Because the mRNA and enzyme activity profiles of CuZnSOD and FeSOD were drastically affected in *lb3* and *lb123* nodules, we examined the expression of three key genes associated with metal homeostasis and senescence: copper chaperone for SOD (*CCS*), cysteine protease 1 (*CYP1*), and ferritin (Fig. 7A, B). CCS is required for Cu delivery to CuZnSOD, and the expression of *CCS* and *CuZnSOD* is regulated by Cu availability (Cohu *et al.*, 2009). *CYP1* is a senescence-associated gene (*SAG*) and a useful marker of nodule senescence (Wang *et al.*, 2023). Ferritin stores Fe in a safe form avoiding Fenton reactions, is regulated by Fe availability, and is responsive to oxidative stress (Ravet *et al.*, 2009). We found that the three genes were highly up-regulated in the *lb123* nodules, with *CYP1* being the most notorious case because its mRNA was undetectable in the WT and all other mutant nodules (Fig. 7A, B). Notably, the *lb3* mutant nodules also showed some changes in ferritin mRNA and protein (Fig. 7B) but these were less pronounced than in the *lb123* mutant. However, the *lb3* nodules showed a similar production of ROS and NO compared with the WT nodules (Fig. 5), and the *CCS* and *CYP1* mRNA levels were not affected (Fig. 7A). Taking together the results shown in Figs 5–7, we conclude that nodules of *lb123*, and to a lesser extent *lb3*, experience nitro-oxidative stress and alterations of metal homeostasis.

### NAC094 is a marker of nodule aging and stress-induced senescence and is required for CYP1 activation even in the absence of nitrate

**Fig. 8.**
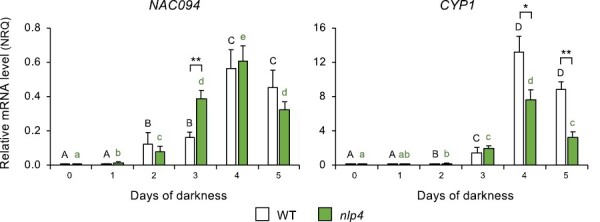
Profiles of *NAC094* and *CYP1* transcripts in nodules of WT and *nlp4* mutant plants of *L. japonicus* during dark stress. Nodulated plants grown without nitrate until 4 wpi were exposed to prolonged darkness for 1–5 d. Transcript levels are expressed as normalized relative quantities (NRQ) using *LjUbiquitin* and *LjeIF4A* as reference genes. Data are means ±SE of 3–12 biological replicates. Means denoted by different uppercase black letters (for treatment comparisons in WT plants) or lowercase green letters (for treatment comparisons in *nlp4* plants) are significantly different according to Duncan’s multiple range test (*P*<0.05). For each treatment, asterisks indicate significant differences between the WT and *nlp4* based on Student’s *t*-test (*P<0.05; **P<0.01).

The contrasting patterns of the various Lbs and Glbs in nodules during aging or after nitrate supply prompted us to further investigate their regulation by means of two loss-of-function mutants, *nlp4* and *nac094*, of transcription factors. We chose them because NLP4 is a master regulator of the symbiotic nitrate response in *M. truncatula* (Lin *et al.*, 2018) and *L. japonicus* (Nishida *et al.*, 2018) and because NAC094 is a positive regulator of nitrate-induced nodule senescence (Wang *et al.*, 2023). Prior to hemoglobin profiling, we determined the responses of both transcription factors in nodules under our experimental setup. First, we quantified *NLP4* and *NAC094* mRNAs during nodule development of WT plants (Supplementary Fig. S4A) as well as in mature nodules of *lb* mutants (Supplementary Fig. S4B). No change in the *NLP4* mRNA level was found under such conditions. In sharp contrast, *NAC094* was highly up-regulated in aging nodules, especially at 10 wpi, and in the *lb123* mutant, although its expression was unaffected in the single and double *lb* mutants. Second, we quantified *NAC094* mRNA in *nlp4* nodules. Following supply of plants at 4 wpi with low or high nitrate for 2 d, the expression level of this gene remained constant in the *nlp4* mutant (Supplementary Fig. S5). Because *NAC094* is up-regulated by high nitrate through NLP4 (Wang *et al.*, 2023), our results may be interpreted in terms of a superior symbiotic tolerance of *nlp4* plants to nitrate (Lin *et al.*, 2018; Nishida *et al.*, 2018). Subsequent experiments included dark stress for comparison with high nitrate because both treatments induce nodule senescence (Escuredo *et al.*, 1996). Plants were grown without nitrate until 4 wpi and then exposed to continuous darkness for 1–5 d under otherwise identical environmental conditions (Fig. 8). In non-stressed nodules, the *NAC094* mRNA was virtually undetectable. In contrast, dark stress led to a progressive up-regulation of *NAC094* in the WT and *nlp4* mutant. Nevertheless, the expression profiles were not identical in the two genetic backgrounds (Fig. 8), which suggests an interplay between NLP4 and NAC094. To confirm that *NAC094* expression was linked to nodule senescence in a nitrate-independent manner, we profiled the senescence marker gene *CYP1*. We found that its expression level followed a similar trend to *NAC094* but was lower in *nlp4* than in the WT after 4 d and 5 d of darkness (Fig. 8). In connection to this finding, we observed that, whereas *CYP1* is strongly activated in WT nodules after 3 d of dark stress, the activation is suppressed in the *nac094* mutant (Supplementary Fig. S6). We conclude that *NAC094* is a general key marker of nodule senescence because it is drastically up-regulated not only in *lb123* nodules but also during developmental and stress-induced senescence. Our results also indicate that *NAC094* is required for activation of *CYP1* in dark-stressed nodules and that this activation also occurs in the absence of nitrate.

### NLP4 and NAC094 modulate hemoglobin expression in a gene-specific manner in response to nitrate

**Fig. 9.**
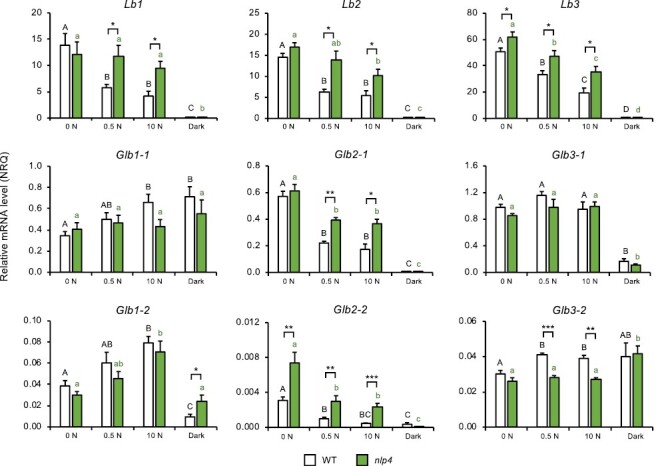
Expression of leghemoglobin (*Lb*) and phytoglobin (*Glb*) genes in nodules of *nlp4* mutants of *L. japonicus* in response to nitrate or dark stress. Nodulated plants were grown without nitrate until 4 wpi and then were treated with 0, 0.5, or 10 mM KNO_3_ for 2 d, or were exposed to continuous darkness for 3 d. Transcript levels are given in normalized relative quantities (NRQ) using the geometric means of *LjUbiquitin* and *LjeIF4A*. Data are means ±SE of 3–11 biological replicates. Means denoted by different black uppercase letters (for treatment comparisons in WT plants) or green lowercase letters (for treatment comparisons in *nlp4* plants) are significantly different from each other according to Duncan’s multiple range test (*P*<0.05). For each treatment, asterisks indicate significant differences between the WT and *nlp4* based on Student’s *t*-test (*P<0.05; **P<0.01; ***P<0.001).

We next profiled expression of *Lb* and *Glb* genes under nitrate or dark stress in the *nlp4* and *nac094* mutants. For clarity and statistical analysis, the results obtained with the two mutants are represented in separate figures but they use the same data of the WT plants. This representation facilitates comparison of each mutant with the WT as well as the response of each genotype to nitrate or dark stress. We found higher mRNA levels of genes encoding Lbs and class 2 Glbs in the *nlp4* mutant than in the WT at low (0.5 mM) and high (10 mM) nitrate concentrations, consistent with the relative nitrate insensitivity of *nlp4* (Fig. 9). In contrast, expression of *Glb1-2* and *Glb3-1* displayed similar levels and nitrate responses in both genotypes. Expression of *Glb1-1* and *Glb3-2* increased with nitrate in the WT but not in *nlp4*. Of these four genes, only *Glb3-2* showed a significantly lower expression in *nlp4* than in WT plants (Fig. 9). These results, together with those of Fig. 2, not only underline a distinct regulation and sensitivity of individual *Lb* and *Glb* genes in response to increasing nitrate, but also evidence transcriptional modulation of most hemoglobin genes by NLP4. The exceptions seem to be *Glb1-2* and *Glb3-1*, which display similar expression trends in the nodules of WT and *nlp4* plants. There were also distinct effects of dark stress on hemoglobin gene expression. Thus, in the WT nodules, the expression of *Glb1-1* increased and that of *Glb3-2* remained unaffected, whereas the expression of all other hemoglobin genes was suppressed or drastically reduced (Fig. 9). However, comparison of control (0 N) and dark-stressed nodules between the WT and *nlp4* reveals some differences in the two backgrounds: *Glb1-1* (up-regulated in the WT), *Glb1-2* (down-regulated in the WT), and *Glb3-2* (up-regulated in *nlp4*) (Fig. 9).

**Fig. 10.**
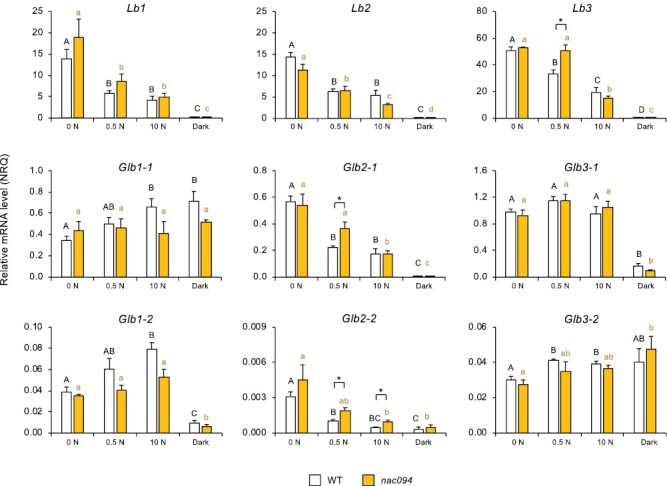
Expression of leghemoglobin (*Lb*) and phytoglobin (*Glb*) genes in nodules of *nac094* mutants of *L. japonicus* in response to nitrate or dark stress. Nodulated plants were treated as indicated in Fig. 9. Transcript levels are given in normalized relative quantities (NRQ) using the geometric means of *LjUbiquitin* and *LjeIF4A*. Data are means ±SE of 3–11 biological replicates. Means denoted by different black uppercase letters (for treatment comparisons in WT plants) or orange lowercase letters (for treatment comparisons in *nac094* plants) are significantly different from each other according to Duncan’s multiple range test (*P*<0.05). For each treatment, asterisks indicate significant differences between the WT and *nac094* based on Student’s *t*-test (*P<0.05).

A similar expression profiling of *Lb* and *Glb* genes was conducted in the *nac094* mutant (Fig. 10). Only a few differences were observed between the nodules of WT and *nac094* mutant plants with nitrate or dark stress. In response to low nitrate, consistent differences were found in the expression of *Lb3* and *Glb2-1* (up-regulated by ~58% in *nac094*). We also detected a major difference of *Glb2-2* expression (up-regulated by ~92% in *nac094*) with low and high nitrate (Fig. 10). These results indicate that *NAC094* modulates the expression of the genes coding for Lb3 and class 2 Glbs, although to variable extents depending on the presence of non-inhibitory or inhibitory nitrate concentrations.

### Conserved NREs and dNREs were identified in hemoglobin genes of *Lotus japonicus*

**Fig. 11.**
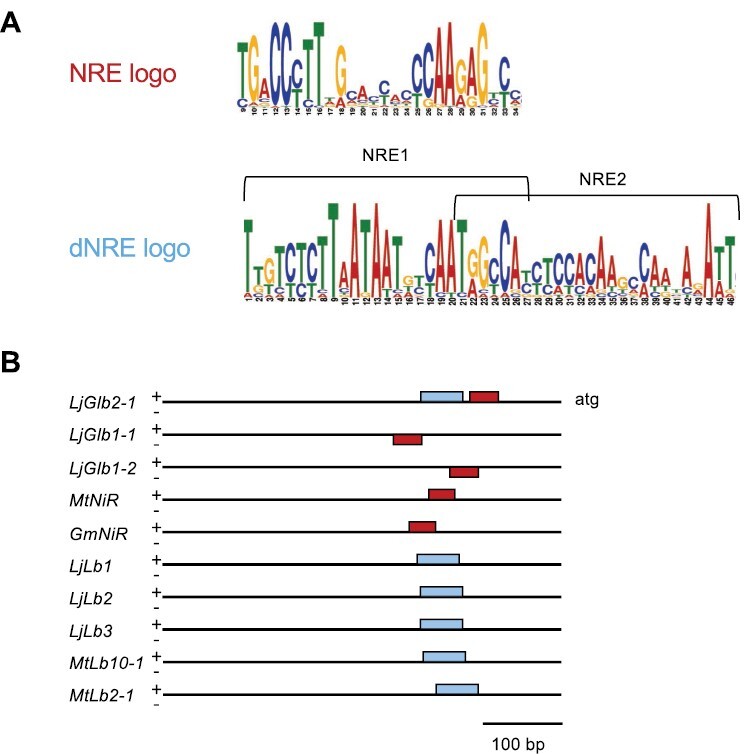
Detection of nitrate-responsive elements (NREs) and double nitrate-responsive elements (dNREs) in the promoters of *L. japonicus* hemoglobin genes using MEME. (A) Sequence logos of the detected NREs and dNREs. (B) Location of NREs and dNREs in hemoglobin promoters. NREs (red boxes) were detected in the promoters of *L. japonicus Glb1-1*, *Glb1-2*, and *Glb2-1* by analyzing them with a large set of nitrite reductase (*NiR*) promoters from different plant species as described in the Materials and methods. dNREs (cyan boxes) were detected in the promoters of *L. japonicus Lb* genes and *Glb2-1* by analyzing them with promoters of 12 *Lb* genes of *Medicago truncatula.* No NRE or dNRE motifs were detected in the promoters of *L. japonicus Glb2-2*, *Glb3-1*, and *Glb3-2.* The gene identifiers for *M. truncatula NiR*, *Glycine max NiR*, *M. truncatula Lb4*, and *M. truncatula Lb11* are Medtr4g086020, Glyma.07G212800, Medtr1g090820, and Medtr5g081030, respectively.

The results shown in Figs 2, 9, and 10 indicate that the expression of hemoglobin genes in nodules of *L. japonicus* is altered in response to different concentrations of nitrate. Promoters of *NiR* genes were previously shown to contain NREs that serve as direct targets for activation of their expression by NLPs (Konishi and Yanagisawa, 2010), whereas dNREs were subsequently identified in the promoters of *Lb* genes and are required for high expression of Lbs in nodules (Jiang *et al.*, 2021). Analysis of the *Lb* and *Glb* promoter sequences using MEME revealed the presence of NREs in the promoters of *Glb1-1*, *Glb1-2*, and *Glb2-1* and of dNREs in the promoters of *Lb* genes and *Glb2-1* (Fig. 11). However, NRE or dNRE motifs were not detected in the promoters of *Glb2-2, Glb3-1*, and *Glb3-2*. The sequences and positions of the NRE and dNRE motifs are provided in Supplementary Tables S2 and S3, respectively.

## Discussion

Hemoglobins are encoded by multigene families and occur as complex mixtures of isoproteins in plant tissues (see reviews by Hoy and Hargrove, 2008; Hill, 2012; Becana *et al.*, 2020). Early studies reported that the proportions of Lb isoproteins in soybean and pea varied with nodule age (Fuchsman and Appleby, 1979; Uheda and Syōno, 1982; Sarath *et al.*, 1986) and that the abundance of two Lbs in mungbean responded oppositely to application of high nitrate (Becana and Sprent, 1989). At that time, the limited availability of genomic sequences, model legumes, and mutants precluded comprehensive and detailed studies of hemoglobin gene regulation. Here we present such a study encompassing the full complement of *L. japonicus* hemoglobins. To this end, we used mutants deficient in Lbs or in transcription factors associated with nitrate sensitivity and nodule senescence (Nishida *et al.*, 2018; Wang *et al.*, 2019, 2023).

A relevant finding of our study was the contrasting transcript profiles of the three *Lb* genes during nodule development (Fig. 1), which suggests a major contribution of Lb1 early in nodule development and a more general role for Lb3 throughout the life span of nodules. This further indicates that *Lb3* is regulated, to some degree, independently of *Lb1* and *Lb2*, despite them all having dNREs. These results contrast with previous studies with *L. japonicus* (Wang *et al.*, 2019) and soybean (Du *et al.*, 2020) in which no major differences were seen in *Lb* expression profiles during nodule development. The discrepancies may be ascribed to the use of different legume species and plant growth conditions; for example, plants were fed on 0.5 mM nitrate by Wang *et al.* (2019) while no nitrate was employed in this study. We also found considerable variations in the expression of *Glb* genes during nodule development. Genes of class 2 Glbs behaved like *Lb1* and *Lb2* whereas those of class 1 Glbs and *Glb3-2* remained essentially unaffected. Notably, *Glb3-1* was down-regulated in old nodules but the expression of the orthologous gene of *M. truncatula* increased by 2-fold under similar conditions (Berger *et al.*, 2020), pointing out differences between legumes in hemoglobin regulation.

We also observed contrasting responses of each hemoglobin gene to nitrate. In particular, it was puzzling that *Lb* gene expression was so sensitive to a nitrate concentration commonly used to promote nodule formation. This was most evident in the *Lb1* and *Lb2* mRNA levels, which were reduced by ~58% at 0.5 mM nitrate (Fig. 2). The distinct nitrate responses place in one group the genes of *Lb* and the unusual class 2 Glbs (down-regulated with 0.5–10 mM) and in another group the genes of class 1 Glbs (up-regulated with 5–10 mM) and class 3 Glbs (up-regulated with 0.5–5 mM). To facilitate comparisons among Lbs and the three Glb classes of *L. japonicus*, we have compiled some important biochemical properties of the proteins along with findings of the present work in Table 1. Although Glb2-1 and Glb2-2 have homology with Lbs and typical class 2 Glbs, they also show some differences. On the one hand, unlike Lbs, which do not contain cysteine (with the exception of Lb1 of *Aeschynomene evenia*; Quilbé *et al.*, 2021), Glb2-1 and Glb2-2 contain cysteine residues and occur in nodules at much lower concentrations (especially Glb2-2) than Lbs. Also, Glb2-1 shows a hexacoordinate heme in the ferric form, does not complement the *lb123* triple mutant, and its deficiency in non-nodulated plants delays growth and causes alterations in the leaf metabolome (Villar *et al.*, 2021). On the other hand, Glb2-1 and Glb2-2 show homology with Lbs, are mainly but not exclusively expressed in nodules, and their expression is strongly down-regulated by nitrate and dark stress, as is the case of Lbs (Table 1). In a recent phylogenetic analysis of ~100 sequences of Lbs, class 1 Glbs, and class 2 Glbs (Becana *et al.*, 2020), we observed that Glb2-1 clusters with *M. truncatula* Lb3 (MtLb3; Medtr1g090810) and that they shared 66% identity and 81% similarity in amino acid sequences. After phenotypic examination of a knockout mutant and biochemical characterization of the protein, we concluded that MtLb3 is an atypical Lb (Villar *et al.*, 2021). It will be worthwhile to investigate whether similar ‘unusual’ Lbs or Glbs with high amino acid identity with *L. japonicus* Glb2-1 or Glb2-2 occur in other legumes.

**Table 1.** Comparison of *Lotus japonicus* hemoglobins

	Leghemoglobins			Class 1 phytoglobins		Class 2 phytoglobins		Class 3 phytoglobins	
Isoproteins	Lb1	Lb2	Lb3	Glb1-1	Glb1-2	Glb2-1	Glb2-2	Glb3-1	Glb3-2
**Protein size** ^*a*^	146	146	147	161	161	152	154	169	168
**Protein mass** ^*b*^	16.0	16.0	16.3	18.6	18.6	17.8	18.4	20.2	20.1
**Cysteine residues**	–	–	–	C8/C78	C79	C65	C51	C159/C166	C159
**Heme coordination (2+)** ^*c*^	Penta	Penta	Penta	Hexa	Hexa	Penta	Penta	Penta	Penta
**Heme coordination (3+)**	Penta	Penta	Penta	Hexa	Hexa	Hexa	Penta	Penta	Penta
**Concentration range**	mM	mM	mM	nM–μM	nM–μM	nM–μM	nM–μM	nM–μM	nM–μM
**Plant organ location** ^*d*^	Nodule	Nodule	Nodule	Nodule	Leaf	Nodule	Nodule	Nodule	Ubiquitous
**Structure** ^*e*^	3-on-3			3-on-3		3-on-3		2-on-2	
**Function** ^*f*^	Transport and delivery of O_2_			Homeostasis of NO		Unknown		Unknown	
**Expression in mature nodules** ^*g*^	8–12	12–15	50–64	0.32–0.36	0.039–0.043	0.42–0.58	0.003–0.006	0.94–1.13	0.020–0.031
**Nodule aging** ^*h*^	↓↓	↓	=	=	=	↓↓	↓↓	↓	=
**Nodule low nitrate response** ^*h*^	↓↓	↓	↓	=	=	↓↓	↓↓	↑	↑
**Nodule high nitrate response** ^*h*^	↓↓	↓	↓	↑↑	↑↑	↓↓	↓↓	↑	↑↑
**Nodule dark stress** ^*h*^	↓↓	↓↓	↓↓	↑↑	↓↓	↓↓	↓↓	↓↓	=
**NREs/dNREs** ^*i*^	dNRE	dNRE	dNRE	NRE	NRE	NRE + dNRE	n.d.	n.d.	n.d.

Data were compiled from Fukudome *et al.* (2016); Larrainzar *et al.* (2020); Wang *et al.* (2019); Villar *et al.* (2021); and this work.

^*a*^ Number of amino acid residues.

^*b*^ Protein molecular mass in kDa including the heme group.

^*c*^ Pentacoordination or hexacordination state of the ferrous (2+) and ferric (3+) forms of the hemoprotein.

^*d*^ Plant organ showing maximal expression.

^*e*^ Hemoglobin classification based on spatial disposition of α-helices relative to the heme: canonical (3/3-fold) or truncated (2/2-fold) tertiary structures.

^*f*^ Main known function, which may not be the single one.

^*g*^ Expression levels in mature nodules (4 wpi) are given as normalized relative quantities.

^*h*^ Up-regulation (↑ moderate; ↑↑ strong), no change (=), or down-regulation (↓ moderate; ↓↓ strong) of gene expression during nodule aging or in nodules of plants supplied with low nitrate (0.5 mM) or high nitrate (5 mM or 10 mM) for 2 d or exposed to dark stress for 3 d.

^*i*^ Presence of nitrate-responsive elements (NREs) or double nitrate-responsive elements (dNREs) in the gene promoters. n.d., not detected.

Previously we had observed an enhanced production of superoxide and H_2_O_2_ in *lb123* mutant nodules (Wang *et al.*, 2019). Here, we show that those nodules undergo nitro-oxidative stress as evidenced by an increased production of ROS and NO (Fig. 5), as well as by major alterations in antioxidant defenses (Fig. 6) and expression of marker genes (Fig. 7). The finding that NO accumulates in the absence of Lbs is a most surprising and novel finding because plants used for cytochemical studies were grown in the absence of nitrate, whereas the only demonstrated pathways of NO production in nodules, namely plant and bacterial nitrate reductases and the mitochondrial electron transport chain, require nitrate (Horchani *et al.*, 2011). An alternative pathway involving an NO synthase-like activity reported in lupine nodules (Cueto *et al.*, 1996) was never verified by direct measurements of NO, nor has a plant enzyme responsible for such activity been identified; hence, we cannot ascertain that the observed accumulation of NO originated through this pathway. We conclude that there exists a nitrate-independent pathway for NO generation that becomes conspicuous in *lb123* nodules. Why is the NO not scavenged to avoid nitro-oxidative stress? Based on our results, we propose that this is due to the lack of Glb1-1 and Lbs (Figs 3, 4) because these hemoproteins are able to convert NO to nitrate *in vitro* through their NO dioxygenase activities (Herold and Puppo, 2005; Rubio *et al.*, 2019). This may also happen *in vivo* as down-regulation of Glb1-1 by 3-fold caused a moderate increase of NO in mature nodules of *M. truncatula* (Berger *et al.*, 2020). Here we found that the *lb123* nodules seem to behave as knockout mutants of Glb1-1 because this protein became almost undetectable on immunoblots (Fig. 4B). However, Lbs may also be important NO scavengers, taking into account that they exhibit NO dioxygenase activity and are present at much higher concentrations than Glb1-1.

The *lb123* mutant nodules also showed some alterations related to Fe homeostasis and/or metabolism. This is supported by two observations. First, ferritin, located in nodule plastids, is transcriptionally activated by oxidative stress (Ravet *et al.*, 2009) and by an increase of intracellular free Fe that may be mediated by NO signaling during nodule senescence (Chungopast *et al.*, 2017). We found that both NO and ferritin (mRNA and protein) accumulate in *lb123* nodules, which is fully consistent with that report (Figs 5, 7). Second, SODs are regulated by the availability of their metal cofactors (Alscher *et al.*, 2002; Moran *et al.*, 2003; Cohu *et al.*, 2009). We found in the mutant nodules that FeSODc is replaced by CuZnSODc, which, together with the up-regulation of *CCS*, suggests some alterations in the availability of Fe and Cu. Also of interest, catalase and APX activities decreased markedly in the *lb123* mutant nodules, which may explain the accumulation of H_2_O_2_. According to a previous RNA-seq analysis of *lb123* nodules (Wang *et al.*, 2019), the transcript levels of APX are not affected. This leads us to conclude that the decline of activities is probably a result of enzyme degradation that takes place during senescence, as evidenced by the activation of *CYP1* and *NAC094* (Fig. 7A; Supplementary Fig. S4B) (Wang *et al.*, 2023). An unexpected observation is that the *lb3* mutant nodules display several alterations resembling those of *lb123* but in an attenuated form. However, the double mutants also lacking Lb3, namely *lb13* and *lb23*, do not show those alterations. We cannot offer an explanation for this, except that the overexpression of *Lb2* and *Lb1*, respectively, compensates at least partially for the deficiency of Lb3.

In our comparison among *L. japonicus* hemoglobins, the detection of NREs and/or dNREs in their gene promoters is especially relevant (Fig. 11; Table 1). The NREs have been identified in the promoters of many well-known nitrate-regulated genes, such as *NiR* (Konishi and Yanagisawa, 2010), whereas the dNREs are required for activation of *Lb* expression in nodules (Jiang *et al.*, 2021). Indeed, the expression of the three *Lb* genes, all of which feature dNREs, was negatively correlated with nitrate concentration (Fig. 2). We found NREs in the promoters of *Glb1-1* and *Glb1-2*, and both genes showed progressively higher levels of expression in nodules with increasing nitrate concentrations. The expression of these genes was not, however, affected in *nlp4* nodules (this work), suggesting that some other NLP regulates them. However, *Glb2-1* also contained an NRE in its upstream sequence but it was repressed, rather than induced, by nitrate. This might be explained by our finding that the *Glb2-1* promoter also has a dNRE (Supplementary Tables S2, S3). In fact, the discovery of both NRE and dNRE in close proximity in the promoter of *Glb2-1* provides further support for our conclusion that Glb2-1 is neither a genuine Lb nor a genuine class 2 Glb (Larrainzar *et al.*, 2020; Villar *et al.*, 2021; this work). Because all known Lbs, except those of *A. evenia* (Quilbé *et al.*, 2021), were recruited for symbiotic function through evolution of class 2 Glbs, we propose that *Glb2-1* is an intermediate gene between Lbs and class 2 Glbs. It is thus not surprising that Glb2-1 also performs non-symbiotic functions, as proven by the altered phenotype of non-nodulated *glb2-1* mutants (Villar *et al.*, 2021) and its expression in flowers (Lotus Expression Atlas, https://lotus.au.dk/). However, *Glb2-1* is consistently down-regulated even with 0.5 mM nitrate, much like *Lb2* (Fig. 2), which suggests that dNRE is prevalent over NRE in its nitrate response. Interestingly, *Glb2-2*, a close homolog of *Glb2-1* (63% identity and 77% similarity in amino acid sequences), harbored neither of these elements. Our data show that the expression level of *Glb2-2* is two orders of magnitude lower than that of *Glb2-1* (Table 1), demonstrating that they are subject to different transcriptional regulation. We failed to detect NREs or dNREs in the promoters of *Glb3-1* and *Glb3-2*, even though both genes showed increased expression in nodules treated with 0.5 mM or 5 mM nitrate (Fig. 2). This could be due to either NREs present outside of the sequence analyzed, or a regulation independent of NLPs. Notably, in *M. truncatula*, expression of class 3 Glbs is activated during nodulation and mycorrhization (Vieweg *et al.*, 2005), suggesting that they could be regulated by symbiotic signaling.

Transcript profiling of *nlp4* and *nac094* mutants also sheds light on the regulation of *Lb* and *Glb* genes in response to nitrate and dark stress. NLP4 is a central regulator of the symbiotic nitrate response and its deficiency confers a certain degree of tolerance of nodule activity to high nitrate (Nishida *et al.*, 2018, 2021). The relative insensitivity of the *nlp4* mutant to nitrate was confirmed by its lower increase of the *NAC094* mRNA level (Supplementary Fig. S5) and enhanced expression of the genes encoding Lbs and class 2 Glbs (Fig. 9) compared with the WT. As NLPs activate nitrate-dependent gene expression (Nishida *et al.*, 2018; Jiang *et al.*, 2021), it seems that the effect of losing NLP4 on the expression of those four hemoglobin genes is indirect, probably through alleviation of nitrate suppression of nodulation. In contrast, the expression profiles of *Glb1-1*, *Glb1-2*, and *Glb3-1* do not differ between the WT and *nlp4* (Fig. 9), and hence the expression of these genes does not require NLP4 for the nitrate response.

In conclusion, we show that hemoglobins of *L. japonicus* are distinctly regulated during development as well as in the nitrate and stress responses of nodules. The nitrate responses fit into two categories: those which showed increased expression in response to nitrate (class 1 and class 3 Glbs) and those which were repressed by nitrate (Lbs and class 2 Glbs). This dicotomy was further supported by the presence of NREs in the promoters of *Glb1-1* and *Glb1-2*, which suggests that they are directly regulated by NLPs as part of the nitrate response, and of dNREs in the promoters of *Lb* genes and *Glb2-1*, which suggests indirect control, via nitrate suppression of nodulation. The use of Lb mutants allowed us to unveil compensatory phenomena between Lb isoproteins and to demonstrate that Lb-deficient nodules experience nitro-oxidative stress as a result of accumulation of ROS and NO, and of alterations in antioxidant defenses and senescence markers. Loss of Lbs also resulted in a large decrease in the content of Glb1-1 in nodules, consistent with an important role for Glb1-1 and Lbs in coping with NO stress. Because the accumulation of NO occurs in the absence of nitrate, a nitrate-independent pathway of NO biosynthesis should exist in nodules. Finally, transcript profiling of *nlp4* and *nac094* mutants showed that NLP4 modulates hemoglobin expression in a gene-specific manner in response to nitrate, whereas loss of NAC094 has a smaller effect.

## Supplementary data

The following supplementary data are available at *JXB* online.

Table S1. *Lotus japonicus* gene accession numbers and primers used for qRT–PCR expression analyses.

Table S2. Sequence and orientation of ‘nitrate-responsive elements’ (NREs) in promoters of hemoglobin genes of *L. japonicus*.

Table S3. Sequence and orientation of ‘double nitrate-responsive elements’ (dNREs) in promoters of hemoglobin genes of *L. japonicus*.

Fig. S1. Scheme of exon–intron composition of the nine hemoglobin genes of *L. japonicus* showing the position of forward and reverse primers used for qRT–PCR analysis.

Fig. S2. Growth phenotype of leghemoglobin mutants of *L. japonicus*.

Fig. S3. Immunoblot of class 3 Glbs of nodules of *glb3-2* and *glb3-1* mutants of *L. japonicus*.

Fig. S4. Expression profiles of *NLP4* and *NAC094* during nodule development and senescence and in leghemoglobin mutants of *L. japonicus*.

Fig. S5. Profiles of *NAC094* transcripts in nodules of WT and *nlp4* mutant plants in response to nitrate.

Fig. S6. Profiles of *CYP1* transcript abundance in nodules of WT and *nac094* plants under dark stress.

erad455_suppl_Supplementary_Tables_S1-S3_Figures_S1-S6

## Data Availability

The data that support the findings of this study are available on request from the corresponding authors.
