# Supplementary material for: Dynamics of hemoglobins during nodule development, nitrate response, and dark stress in Lotus japonicus
Source: J Exp Bot. 2023 Nov 17;75(5):1547–64. doi: 10.1093/jxb/erad455 (PMC10901204; doi:10.1093/jxb/erad455)
Supplement: erad455_suppl_Supplementary_Tables_S1-S3_Figures_S1-S6 [file erad455_suppl_supplementary_tables_s1-s3_figures_s1-s6.pdf]

Table S1. *Lotus japonicus* gene accession numbers and primers used for qRT-PCR expression analyses.

Gene	Gene ID (MG-20)	Gene ID (Gifu)	Forward sequence (5'→3')	Reverse sequence (5'→3')	Primer efficiency (%)
Ubiquitin	5g0011248	5g1v0317900	TTCACCTTGTGCTCCGTCTTC	AACAACAGCACACACAGACAA	94
ATP-synthase	5g0006532	5g1v0332700	AACACCACTCTCGATCATTTCTCTG	CAATGTCGCCAAGGCCCATGGTG	93
EIF4A	6g0009642	6g1v0242500	AGAGGGTTTAAAGATCAAAT	ATGTCAATTCATCACGTTTT	92
Lb1	5g0024797	5g1v0024700	TGCAATTAAGAAGGCAATG	GGAATACATATGGTTATAGAAATA	97
Lb2	5g0024797	5g1v0024900	TGAAGTTGTATCATCGGAGGAC	GTAGAACAGAACACTATTGCTAGG	93
Lb3	5g0002699	5g1v0046500	TGCTGCCACTCAACTCGTAG	AAGTCATCACTCCATTCGTCCG	88
Glb1-1	3g0017143	3g1v0504500	TTGAGGTTACAAAGTTTGCACTAC	TGCATTCTTCATCTCTGGTGAC	90
Glb1-2	3g0010114	3g1v0504600	CAGTGCCATCATAGCTGAAA	TATTGAAACTGAGAGCAAAGGG	99
Glb2-1	5g0004398	5g1v0253250	CTCAGCCCTTCAACTAAGAG	CTTTAAGCACCAGGAAATGGG	97
Glb2-2	5g0019740	5g1v0253200	GATGGAGTGCCTCACAATAATC	GTAGCATCCGTCACATCTATTTC	82
Glb3-1	1g0018927	1g1v0398700	AGGTGGTTACATCACATGCAA	CATGGACTGGGTGCATTCTT	91
Glb3-2	1g0018426	1g1v0172000	GCAACAAGCATTAGACAGTACTC	TTCTTTAGCTCATTTCCAGCC	98
CuZnSODc	1g0026057	1g1v0777100	TGAACAATGGTGAAGGCTGTG	CCTTGACATTATCGCTGCTGC	97
CuZnSODp	3g0015755	3g1v0484800	GATGGAGTCGCAGAGGCAAC	GGCCAGTGAGTGGAATCTGAC	99
MnSOD	1g0015160	1g1v0308600	CCTCAGCCGTCGTTAAGCTC	GACCTCCGCCATTGAATTTG	99
FeSODc	5g0005373	5g1v0257700	GGTTGCAAAATCTCAGCTTGC	GCTGGAGTGACTTTTGGACCC	90
FeSODp	6g0012455	6g1v0018800	TCGCCATCCACTTCCACTACT	GAAACGCGGAAGGTGATGAT	90
Ferritin	3g0027522	3g1v0197600	AAAGAGGGCATGCAGAGAAA	CCTTCCACCACGAATGTTCT	100
CCS	4g0009345	4g1v0210500	CATTAAGGGAGTGAAGAACGTG	CGAACCGAGTATCCTAACAACC	100
CYP1	3g0024815	3g1v0249300	CTGAAGCTCAATATCCCTATCAAG	CTGGCATCAATTGCTACAGAG	94
NLP4	5g0025579	5g1v0299300	CTGGGAGTCTTAAAGATGCTG	TTAACCTTGTTGATCTTCCGAG	97
NAC094	2g0026112	2g1v0259200	CCTCAGGACATGCAACCAAAC	GATGCCAAAGCAGACTTCCAAG	87

Gene identifiers (IDs) for ecotype MG-20 were retrieved from Li *et al.* (2020) and are given, for simplicity, without the prefix "Lj". In this genome version, *Lb1* and *Lb2* have the same annotation. For further reference to genome MG-20 v3.0, the IDs for hemoglobin genes are as follows: *Lb1* (Lj5g3v0035290.2), *Lb2* (Lj5g3v0035290.1), *Lb3* (Lj5g3v0465970), *Glb1-1* (Lj3g3v3338170), *Glb1-2* (Lj3g3v3338180), *Glb2-1* (Lj5g3v1699110), *Glb2-2* (Lj5g3v1699120), *Glb3-1* (Lj1g3v2035270), and *Glb3-2* (Lj1g3v0948590). Gene IDs for ecotype Gifu B-129 were retrieved from Kamal *et al.* (2020) and are given, for simplicity, without the prefix "LotjaGi".

Additional references:

Kamal N, Mun T, Reid D, Lin JS, Akyol TY, Sandal N, Asp T, Hirakawa H, Stougaard J, Mayer KFX, *et al.* 2020. Insights into the evolution of symbiosis gene copy number and distribution from a chromosome-scale *Lotus japonicus* Gifu genome sequence. DNA Research 27**,** 1–11.

Li H, Jiang F, Wu P, Wang K, Cao Y. 2020. A high-quality genome sequence of model legume *Lotus japonicus* (MG-20) provides insights into the evolution of root nodule symbiosis. Genes 11, 483.

Table S2. Sequence and orientation of 'nitrate-responsive elements' (NREs) in promoters of hemoglobin genes (*Glbs*) of *L. japonicus*.

Gene	Position	Strand	*p*-value (α=0.05)	NRE (5'-> 3')
Glb1-1	-156 to -194	-	1.82 E-08	ATGAAAGTTGGCCCTTCATGGCAATGAAGAGTTTATTAT
Glb1-2	-90 to -128	-	3.84 E-07	AGATTCAGTGTCCCTTTGTGACAATGGAGAGTTATTTTA
Glb2-1	-73 to -111	+	7.03 E-08	ATCAACTTCAACTCTTGGCACTCTCCAAAGCCTCCTCCT

Table S3. Sequence and orientation of 'double nitrate-responsive elements' (dNREs) in promoters of hemoglobin genes (*Lbs* and *Glbs*) of *L. japonicus.*

Gene	Position	Strand	*p*-value (α=0.05)	dNRE (5'-> 3')
Lb1	-130 to -188	+	5.26 E-16	TTATCTCTTAATAATGCCAATGGCCAGCCATCCACAGACTAAAAATTATTCCAATCACC
Lb2	-127 to -185	+	4.20 E-16	TTGTCTCTTAATAAAACCAATGGCCAGCCATCCACCGATGCAGAAATTTTCCAATTAAT
Lb3	-127 to -185	+	3.95 E-17	TTGTCTCTTAATAATGCTAATAGCCATGCACCACACACCAGAAATTCTTCCAATCACGA
Glb2-1	-121 to -179	+	1.00 E-14	TCTTTCTCTCAGAATATTCTTACCACCCTCCACAAGCCAAGAGATGCAATAAAAAACAA

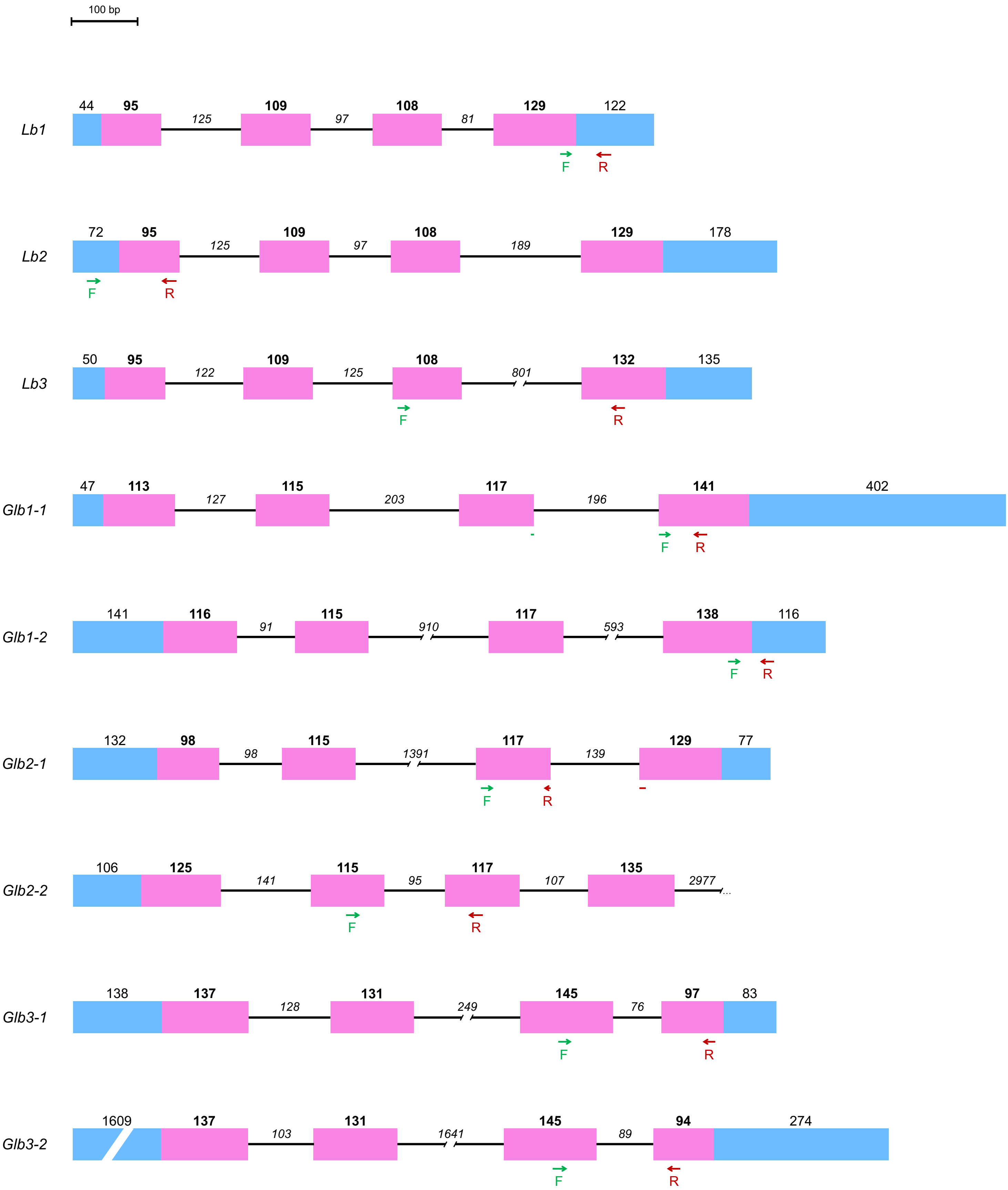

Fig. S1. Scheme of exon-intron composition of the nine hemoglobin genes of *L. japonicus* showing the position of forward (F) and reverse (R) primers used for qRT-PCR analysis. The coding regions of exons are depicted in magenta boxes, UTRs in blue boxes, and introns in black lines. Numbers are lengths in bp and are drawn to scale.

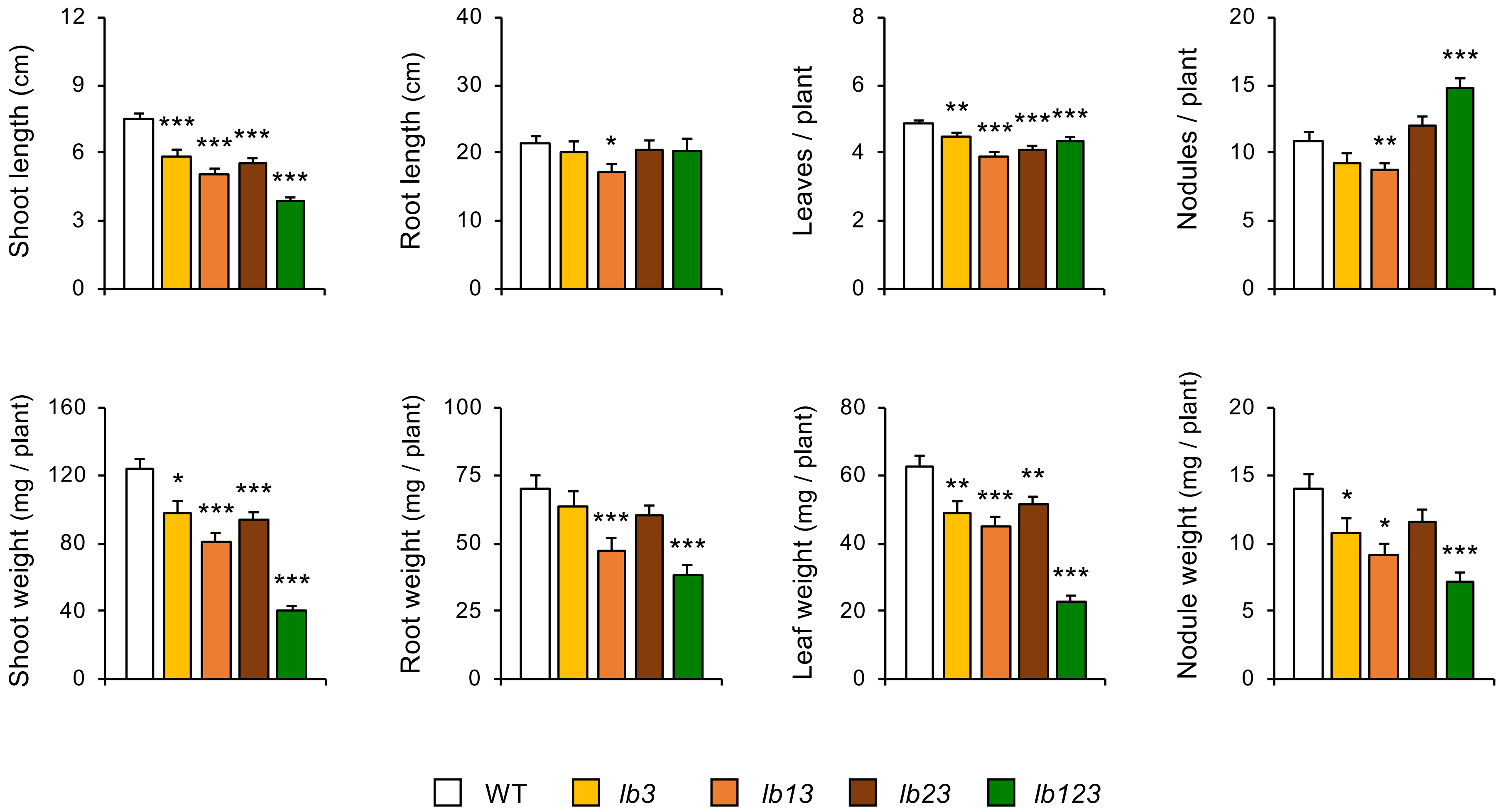

Fig. S2. Growth phenotype of leghemoglobin mutants (*lb*) of *L. japonicus*. Nodulated plants deficient in one (*lb3*), two (*lb13* and *lb23*), or all three (*lb123*) Lbs were grown until 4 wpi without nitrate supply. Data are means ±SE of 24 plants per genotype. Asterisks denote significant differences relative to the wild-type (WT) plants based on Student's *t*-test (*, P<0.05; **, P<0.01; ***, P<0.001).

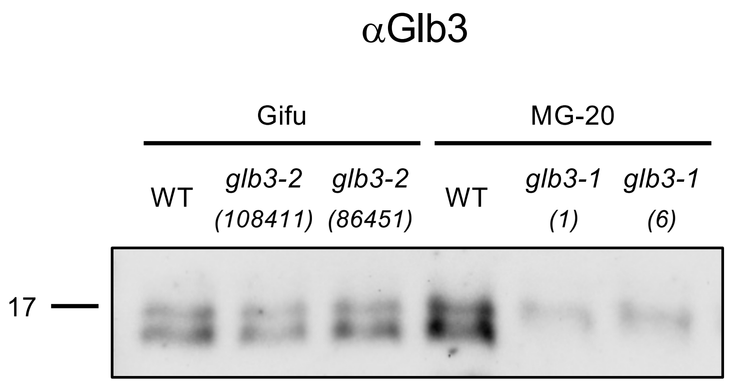

Fig. S3. Immunoblot of class 3 Glbs of nodules of *glb3-2* and *glb3-1* mutants of *L. japonicus*. Nodulated plants were grown without nitrate until 4 wpi. The *glb3-2* mutants were obtained from the *LORE1* collection (Lotus Base, https://lotus.au.dk; Villar *et al.,* 2021) and the *glb3-1* mutants were generated by us using CRISPR/Cas9 following published protocols (Wang LX *et al*., 2016). For each mutant, nodules of two independent homozygous mutant lines were used: 86451 and 108411 (formerly 30086451 and 30108411) for *glb3-2* and lines 1 and 6 for *glb3-1*. Two different wild-type (WT) nodules were used depending on the *L. japonicus* ecotype used to generate the mutants: Gifu (*glb3-2* mutants) and MG-20 (*glb3-1* mutants). SDS-PAGE gels were loaded with 20 μg of protein per lane. Proteins were detected with an affinity-purified polyclonal antibody raised against *L. japonicus* Glb3-2 (αGlb3). The primary and secondary antibodies were used at dilutions of 1:1000 and 1:80000, respectively. The molecular mass (kDa) is indicated on the left.

Additional references:

Villar I, Rubio MC, Calvo-Begueria L, *et al.* 2021. Three classes of hemoglobins are required for optimal vegetative and reproductive growth of *Lotus japonicus*: genetic and biochemical characterization of LjGlb2-1. Journal of Experimental Botany 72, 7778–7791.

Wang LX, Wang L, Tan Q, Fan Q, Zhu H, Hong Z, Zhang Z, Duanmu D. 2016. Efficient inactivation of symbiotic nitrogen fixation related genes in *Lotus japonicus* using CRISPR-Cas9. Frontiers in Plant Science 7, 1333.

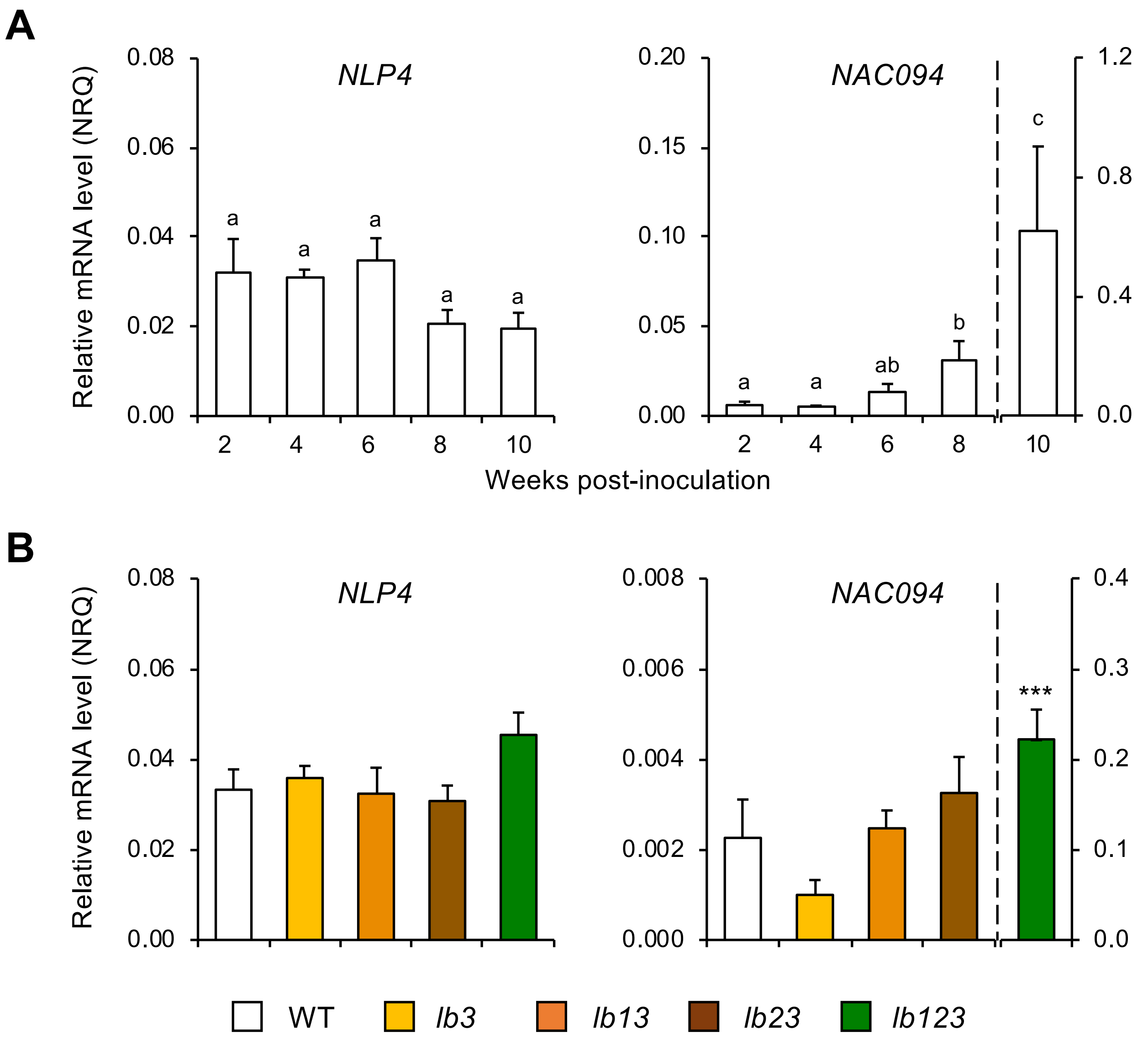

Fig. S4. Expression profiles of *NLP4* and *NAC094* during nodule development and senescence (A) and in leghemoglobin (*lb*) mutants (B) of *L. japonicus*. Transcript levels are expressed in normalized relative quantities (NRQ) using *LjUbiquitin* and *LjeIF4A* (A) or *LjUbiquitin* and *LjATP synthase* (B) as reference genes. Data are means ±SE of 3–5 biological replicates. In A, means denoted by the same letter do not significantly differ (P<0.05) according to Duncan's multiple range test. In B, only the *NAC094* mRNA level in *lb123* nodules was significantly higher than in the WT nodules based on Student's *t*-test (***P<0.001). Neither of the other mean comparisons differed significantly at P<0.05.

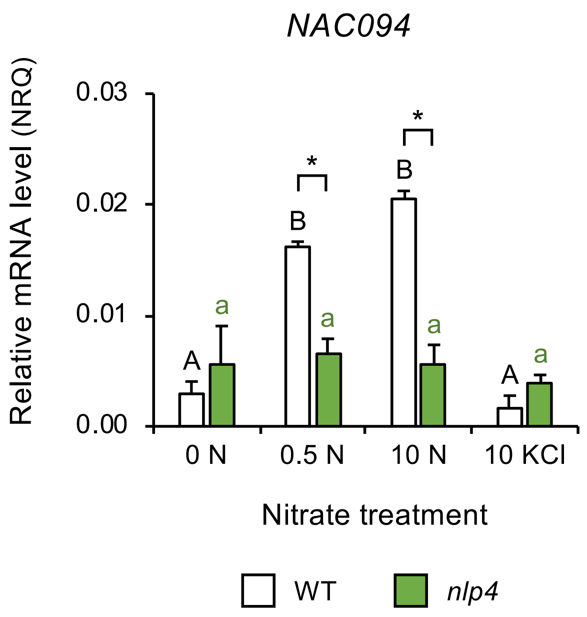

Fig. S5. Profiles of *NAC094* transcripts in nodules of WT and *nlp4* mutant plants in response to nitrate. Nodulated plants were grown without nitrate until 4 wpi and were then treated with 0, 0.5, or 10 mM KNO_3 for 2 d. Control plants supplied with 10 mM KCl for 2 d in place of nitrate were included. Transcript levels are expressed as normalized relative quantities (NRQ) using *LjUbiquitin* and *LjeIF4A* as reference genes. Data are means ± SE of 3–7 biological replicates. Means denoted by different uppercase black letters (for treatment comparisons in WT plants) or lowercase green letters (for treatment comparisons in *nlp4* plants) differ significantly from each other according to Duncan's multiple range test (P<0.05). For each treatment, asterisks indicate significant differences between WT and *nlp4* based on Student's *t*-test (*, P<0.05).

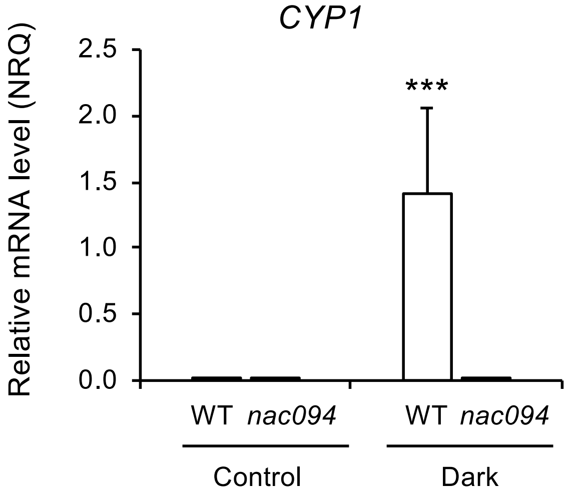

Fig. S6. Profiles of *CYP1* transcript abundance in nodules of WT and *nac094* plants under dark stress. Nodulated plants were grown without nitrate until 4 wpi and were then exposed to continuous darkness for 3 d. Transcript levels are expressed as normalized relative quantities (NRQ) using *LjUbiquitin* and *LjeIF4A* as reference genes. Data are means ±SE of 3–5 biological replicates. Asterisks indicate significant differences between WT and *nac094* based on Student's *t*-test (***, P<0.001).
